# Supplementary material for: Waterborne Polyurethane Acrylates Preparation towards 3D Printing for Sewage Treatment
Source: Materials (Basel). 2022 May 5;15(9):3319. doi: 10.3390/ma15093319 (PMC9104063; doi:10.3390/ma15093319)
Supplement: Supplementary file 1 [file materials-15-03319-s001.zip › materials-1706880-supplementary.pdf]

Supporting Information

Waterborne Polyurethane Acrylates Preparation toward 3D Printing for Sewage Treatment

Kunrong Li[1,2], Yan Li[3], Jiale Hu[1,2], Yuanye Zhang[1,2], Zhi Yang[2], Shuqiang Peng[2], Lixin Wu[2*], Zixiang Weng[2,*]

1 College of Chemistry, Fuzhou University, Fuzhou, Fujian 350108, People's Republic of China; likunrong@fjirsm.ac.cn (K.L.); hujiale@fjirsm.ac.cn (J.H.)
2 Fujian Institute of Research on the Structure of Matter, Chinese Academy of Sciences, Fuzhou, Fujian 350002, People's Republic of China; zhangyuanye@fjirsm.ac.cn (Y.Z.); yangzhi@fjirsm.ac.cn (Z.Y.); pengshuqiang@fjirsm.ac.cn (S.P.)
3 School of Ecological Environment and Urban Construction, Fujian University of Technology, Fuzhou, Fujian, 350118, People's Republic of China; yanli_amy@fjut.edu.cn (Y.L.)
* Correspondence: lxwu@fjirsm.ac.cn (L.W); wzx@fjirsm.ac.cn (Z.W)

Table S1 Molar radio in WPUA.

Samples \ Reagents	IPDI	Diol	DMPA	TEA	HEA	EDA	L-Lys
**WPUA-E**	3.52	1	1	1	1.52	0.76	0
**WPUA-L**	3.52	1	1	1.76	1.52	0	0.76

Table S2 Formulation of **artificial sewage**. (pH = 7.5)

**Reagents**	**$(NH_4)_2SO_4$**	**$Na_3C_6H_5O_7$**	**Na_2HPO_4 $12H_2O$**	**KH_2PO_4**	**$MgSO_4$ $7H_2O$**	**NaCl**	**Trace elements**
**Content (g/L)**	0.25	1.3	1.26	0.5	0.2	30.0	4.0

Formulation of **trace elements**. (pH = 6)

**Reagents**	**EDTA**	**$ZnSO_4$**	**$(NH_4)_6Mo_7O_{24}$ $4H_2O$**	**$MnCl_2$ $4H_2O$**	**$FeSO_4$ $7H_2O$**	**$CoCl_2$ $6H_2O$**	**$CuSO_4$ $5H_2O$**	**$CaCl_2$**
**Content (g/L)**	50	2.2	1.1	5.06	5.0	1.61	1.57	5.5

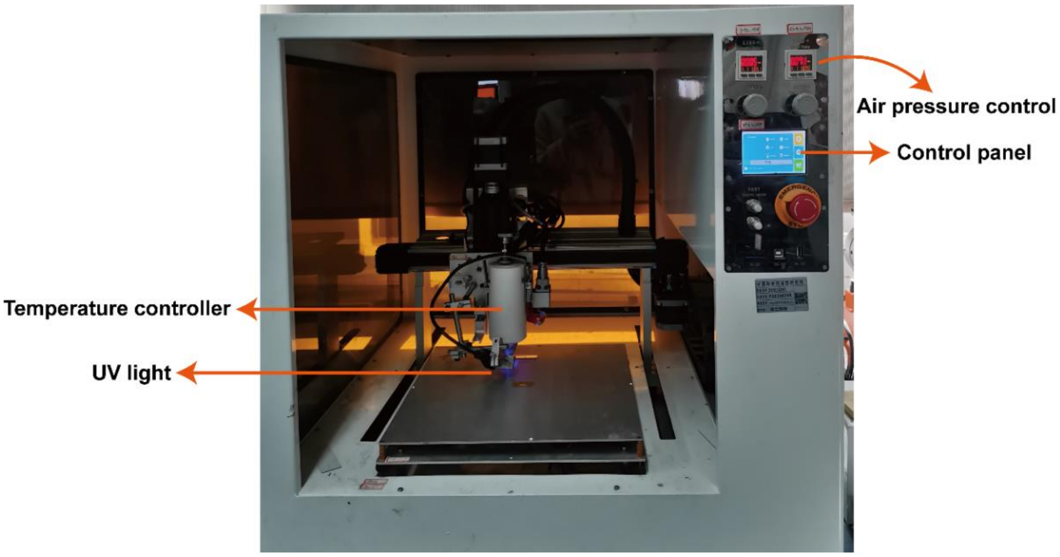

Figure S1 DIW machine used in this study.

Table S3 The details for the determination of NH^{4+}.

**Solution**	**Formulation**
**NaSal solution**	0.06 g Sodium nitroprusside + 8.5 g Sodium salicylate + 100 ml H_2O
**Sodium dichloroisocyanurate (SDIC)**	2.4 g NaOH + 0.5 g SDIC + 100 ml H_2O

The method details are as follows: First, 150 ul Samples, 30 dd H_2O, 60 ul NaSal solution, and 60 ul SDIC were added in a 96-well microplate. These mixed solutions were subsequently incubated at room temperature for 30 mins. The absorbance was measured on an ultraviolet spectrophotometer at the wavelength 660 nm.

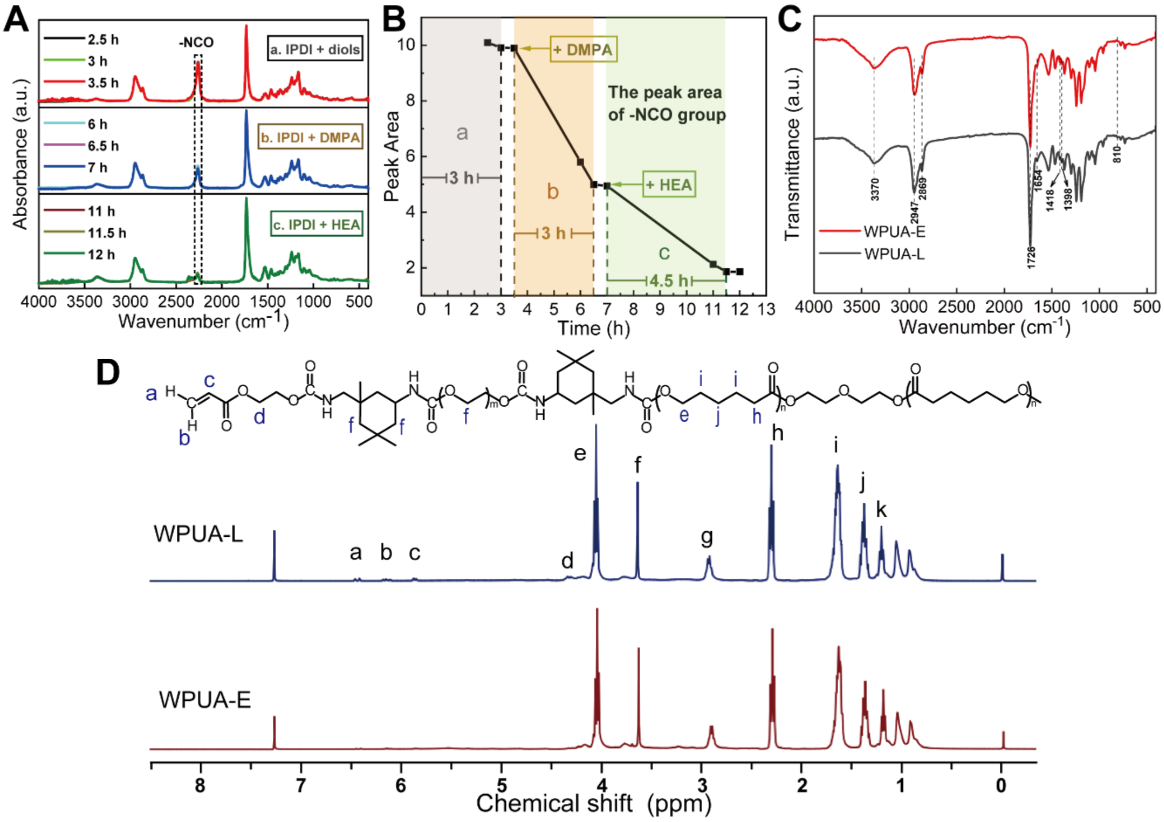

Figure S2 **(A)** FTIR spectra of each reaction step of the synthetic WPUA. **(B)** The peak area changes of −NCO group during WPUA synthesis. **(C)** FTIR spectra of synthesized WPUAs. **(D)** The ^{1}H NMR of WPUAs.

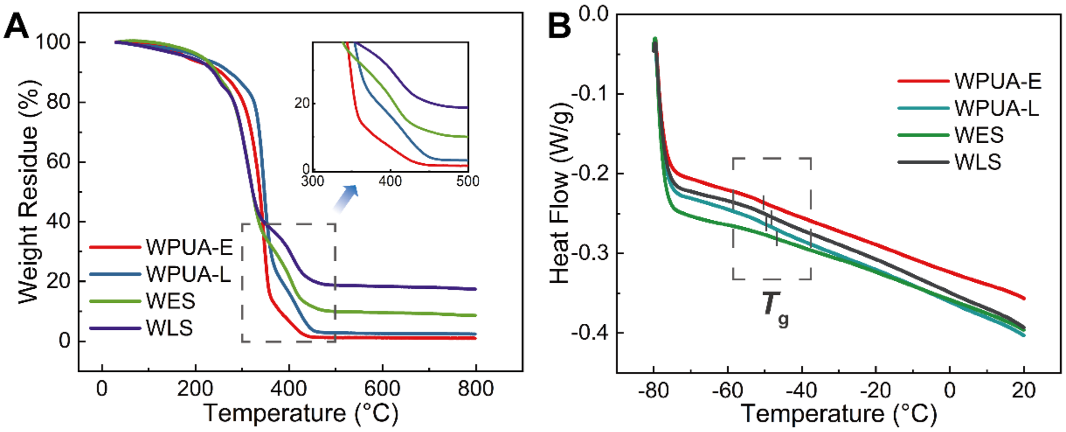

Figure S3 **(A)** TGA and **(B)** DSC curves of WPUA−E, WPUA−L, WES and WLS.

The thermal properties of films

The thermal stability of films was tested by the thermogravimetric method. The test results are shown in Fig. S3A. It can be seen that the decomposition of all films can be divided into two stages. The degradation at the first stage ranged from 250 to 350 °C is mainly caused by the decomposition of polyurethane and polyurea bonds. While the second stage started from 300 to 450 °C which was primarily driven by the rapid degradation of the soft segment. The degradation after 400 °C may be caused by the degradation of the remaining hard segment, and the slight difference may be due to the different choice of polyamine chain extenders. The WES and WLS samples with SA were degraded at a lower temperature than the corresponding samples without SA (WPUA-E and WPUA-L). There would be about 8.6% and 17.4% residue at the later stage due to the formation of sodium oxide, while the residue of pure WPUA-E and WPUA-L films were only about 0.9% and 2.4%, respectively. Also, the glass transition temperature of all as-synthesized WPUAs were evaluated by DSC, and corresponding results are compiled in Fig. S3B. The T_g of WPUA-E, WPUA-L, WES, and WLS were -50.54 °C, -48.42 °C, -46.87 °C and -48.46 °C, respectively. All T_g were lower than -45 °C, indicating that all samples exhibited good viscoelasticity at room temperature.

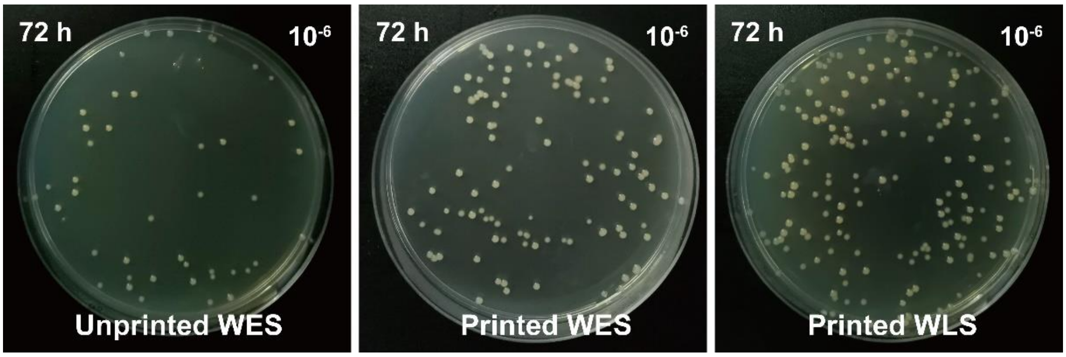

Figure S4 Intuitive diagram of 72 h dilution to 10^{-6} plate counting method.
